# MV-Flow and LumiFlow: new Doppler tools for evaluating the microvasculature of the fetal head

**DOI:** 10.1590/0100-3984.2020.0137

**Published:** 2021

**Authors:** Alessandro Giuffrida, Alberto Borges Peixoto, Edward Araujo Júnior

**Affiliations:** 1 Center of Prenatal Diagnosis G2 Medica, Catania, Italy.; 2 Hospital Universitário Mário Palmério, Universidade de Uberaba (Uniube), Uberaba, MG, Brazil.; 3 Department of Obstetrics and Gynecology, Universidade Federal do Triângulo Mineiro (UFTM), Uberaba, MG, Brazil.; 4 Department of Obstetrics, Escola Paulista de Medicina da Universidade Federal de São Paulo (EPM-Unifesp), São Paulo, SP, Brazil.; 5 Medical Course, Universidade Municipal de São Caetano do Sul/Campus Bela Vista, São Paulo, SP, Brazil.

The microcirculation plays a crucial role in the interaction between blood and tissue, in physiological as well as pathological processes, ensuring fetal and maternal well-being^(1)^. The assessment of the vascularization of the fetal brain is crucial for the evaluation of fetal growth as well as of central nervous system development^(2)^.

Power Doppler has been used in order to study vascular anatomy, especially in areas of low-velocity blood flow^(3)^. However, even when power Doppler is used, it is challenging to evaluate the microvasculature in some regions of the body^(4)^. The MV-Flow and LumiFlow tools, included with the HERA W10 ultrasound system (Samsung Co., Seoul, South Korea), are newly developed Doppler technologies for visualizing the microvasculature and slow-flow connections that cannot be visualized with conventional techniques^(4)^.

Conventional power Doppler shows the magnitude of the color flow rather than the Doppler frequency signal, using filters to remove clutter and motion artifacts, thus resulting in the loss of low-flow components. Therefore, blood flow at a velocity below the constant (< 200 Hz) cannot be detected by conventional techniques^(5)^. The MV-Flow tool allows the visualization of low-velocity flow in the microvasculature of the structures studied, providing greater diagnostic accuracy in comparison with color Doppler and conventional power Doppler. In addition, it offers better sensitivity, better resolution, and higher frame rates.

The LumiFlow tool employs a two-dimensional technology that uses a post-processing tool to give an image a three-dimensional appearance. LumiFlow allows frozen and real-time images of color Doppler, S-flow power Doppler, tissue Doppler, and MV-Flow to create the three-dimensional appearance. The difference in flow velocity within a vessel is variously represented by different colors, corresponding to degrees of brightness. The region with the highest flow velocity (the center of the blood vessel) is represented by a lighter color, whereas the region with the lowest flow velocity (the periphery of the vessel) is represented by a darker color. Therefore, higher flow speed is expressed as areas of greater brightness and lower flow speed is expressed as dimmer, more shaded areas within the image.

To our knowledge, there has been only one study using MV-Flow and LumiFlow to evaluate the venous anatomy of the central nervous system^(4)^. Dall’Asta et al.^(4)^ evaluated the position of the confluence of the venous sinuses, also known as the torcular herophili, to determine the position of the tentorium in 99 pregnancies, including one case of open spina bifida, one case of Dandy-Walker malformation, and two cases of Blake’s pouch cysts.

**Figure 1.**
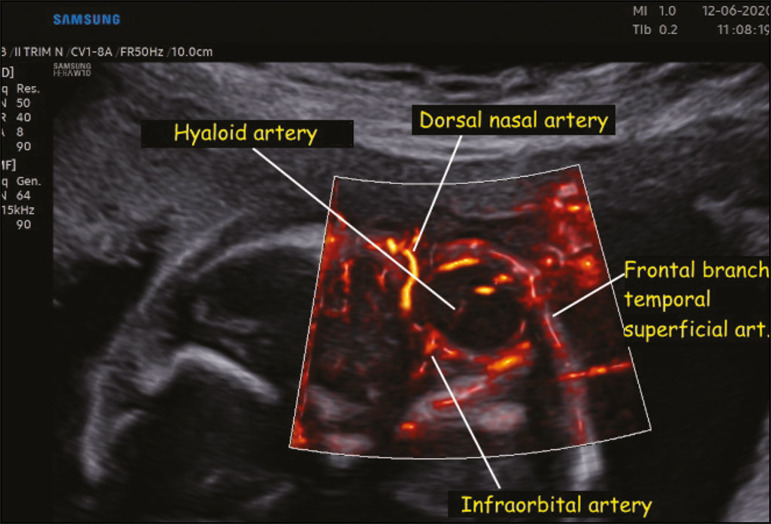
Coronal view of the fetal face, obtained with MV-Flow and LumiFlow at 28 weeks of gestation, showing the orbital arteries. art., artery.

**Figure 2.**
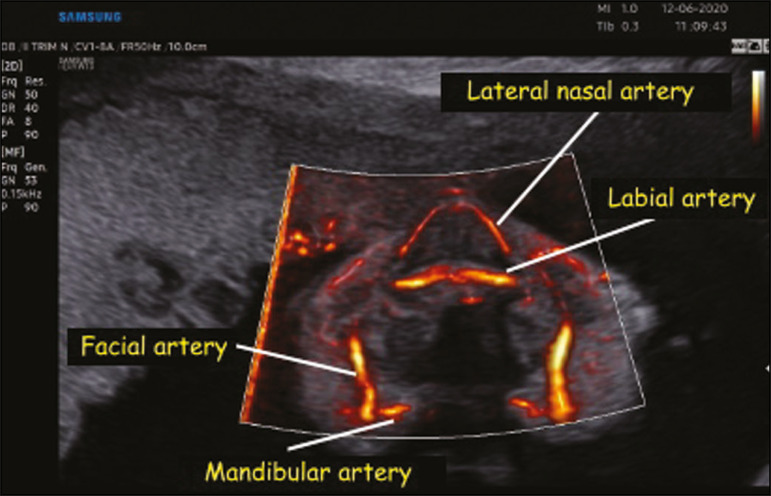
Coronal view of the fetal face at the level of the alveolar portion of the palate, obtained with MV-Flow and LumiFlow at 28 weeks of gestation, showing the facial, labial, nasal, and mandibular arteries.

**Figure 3.**
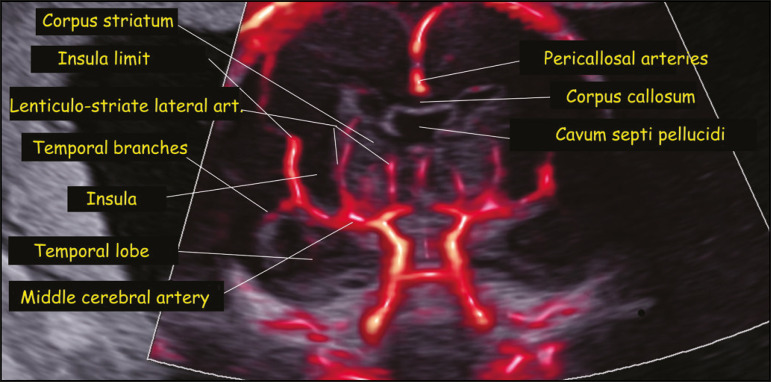
Coronal view of the fetal central nervous system at the level of the corpus callosum, obtained with MV-Flow and LumiFlow at 22 weeks of gestation, showing the cerebral microcirculation. art., artery.

In the present study, MV-Flow and LumiFlow were both able to demonstrate the flow in small vessels of the fetal head, such as those in the palate, orbits, and brain, which were not described in previous studies using color or power Doppler. In the assessment of the orbital region, both techniques were able to provide visualization of the hyaloid, infraorbital, and dorsal nasal arteries (Figure 1). In the assessment of the palatal region, both techniques made it possible to visualize the facial, mandibular, labial, and lateral nasal arteries (Figure 2). In the assessment of the circle of Willis, both techniques were able to identify very thin vessels such as the lateral lenticulostriate artery (Figure 3).

In summary, we believe that these new Doppler techniques constitute important tools for demonstrating the normal or abnormal development of the fetal micro vasculature.
